# The Indirect Effect of the Frailty of Dependent Elderly People Needing Hospitalization on Their Informal Caregivers' Anxiety

**DOI:** 10.7759/cureus.25144

**Published:** 2022-05-19

**Authors:** Ioannis Vrettos, Fotios Anagnostopoulos, Panagiota Voukelatou, Stefani Panayiotou, Andreas Kyvetos, Alexandra Tsigkri, Georgios Boulmetis, Dimitris Niakas

**Affiliations:** 1 Department of Internal Medicine, General and Oncology Hospital of Kifissia “Agioi Anargyroi”, Athens, GRC; 2 School of Social Sciences, Hellenic Open University, Patras, GRC; 3 Department of Psychology, Panteion University of Social and Political Sciences, Athens, GRC; 4 School of Health Sciences, Department of Health Economics, National and Kapodistrian University of Athens, Athens, GRC

**Keywords:** caregiver, anxiety, objective burden, subjective burden, frailty, elderly persons, clinical frailty scale

## Abstract

Background

A high prevalence of anxiety symptoms has been identified among the caregivers of disabled older people. The aim of the study was to explore the relationships between objective burden (intensity of care and burdensome characteristics of the care recipient, like frailty status), caregiver characteristics, subjective burden, and anxiety in a sample of informal caregivers caring for hospitalized elderly patients.

Methods

In this cross-sectional study, patients' and their informal caregivers' characteristics were recorded for 311 patient-caregiver dyads. Subjective caregiver burden and caregivers' anxiety were assessed by using the Zarit Burden Interview and the Hospital Anxiety and Depression Scale (HADS), respectively. Correlation coefficients and path analysis were used to examine the relationship between variables. Caregivers' anxiety was considered as the outcome variable. Caregivers' subjective burden was entered as a mediator between caregiver characteristics-objective burden and anxiety. An objective burden was measured based on the care needs of the dependent elderly (frailty status, cognitive impairment, comorbidity, independence in activities of daily living, behavioral problems, hours spent on caregiving, and duration of caregiving).

Results

Abnormal anxiety symptoms (HADS score 11-21) were reported by 92 caregivers (29.6%). Borderline cases (HADS score 8-10) were 66 caregivers (21.2%). A mild, moderate, or severe subjective burden was recorded for 113 (36.3%), 100 (32.2%), and 26 (8.4%) caregivers, respectively. The female gender of the caregiver, the spousal relationship with the patient, and the subjective burden were directly related to higher levels of caregivers' anxiety. A subjective burden was found to be a significant mediator in the relationship between duration of caregiving, patients' frailty status, caregiver gender, patients' comorbidity, and caregivers' anxiety.

Conclusion

Among the risk factors for caregivers' anxiety, the frailty status of the patient is probably the only modifiable factor via interventions targeting frailty reversion or reduction.

## Introduction

Caring for disabled older people is burdensome and stressful and contributes to psychiatric morbidity in their informal caregivers [1]. Previous studies have identified a high prevalence of anxiety symptoms among caregivers [2,3]. Several factors have been associated with caregivers' anxiety that can be categorized into two groups: factors related to the care recipient, such as the impairment in activities of daily living (ADL) [4], the behavioral problems, the cognitive impairment [5], and the lower physical function [6] of the care recipient and factors related to caregivers such as the spousal relationship with care-recipients [2], being a caregiver living with the care-recipient [4], being a female caregiver [2,4], poor reported health of caregiver [4,6], increased caregivers' age [5] and the subjective caregiver burden [6-8].

The precise relationship between caregivers' burden and caregivers' anxiety is complex. Firstly, caregivers' burden has several common risk factors with anxiety [9]. Secondly, according to one theory, caregiving itself is not stressful, and the appearance of stress is triggered by the caregiver's negative perception of the caregiving situation. More specifically, it has been mentioned that caregiver stress is related both to objective stressors (such as physical function, cognitive impairment, behavioral problems, and intensity of care) and subjective stressors, like subjective burden [10]. A previous study addressing the relationship between anxiety, subjective burden, and objective burden in caregivers of dependent older individuals demonstrated that, independently of the objective burden, the subjective burden is a risk factor for anxiety that mediates the effects of the objective burden on anxiety [8].

The aim of our study was to add to the literature findings regarding the relationships between objective burden, caregiver characteristics, subjective burden, and anxiety in a sample of caregivers caring for hospitalized elderly patients. 

For the purpose of the study, six hypotheses were formulated and tested based on existing literature:

Hypothesis 1: Objective burden (intensity of care and burdensome characteristics of care recipient) is related to subjective burden.

Hypothesis 2: Caregiver characteristics (gender, age, etc.) are related to subjective burden.

Hypothesis 3: Objective burden is related to anxiety.

Hypothesis 4: Caregiver characteristics are related to anxiety.

Hypothesis 5: Subjective burden is related to anxiety.

Hypothesis 6: Subjective burden mediates the relationship between caregiver characteristics, objective burden, and anxiety.

## Materials and methods

Sample, setting, and data collection

From September 2020 to December 2021, a cross-sectional study was conducted in the General and Oncological Hospital of Kifissia "Agioi Anargyroi" among the primary caregivers of dependent elderly patients who were consecutively admitted through the emergency department. As defined by Spillman et al. [11], a caregiver is someone who assisted in at least two categories of the caregiving role (self-care and mobility activities, household activities, transportation, health or medical care activities, and interactions with the health care system and providers). The following criteria were used to determine eligibility: 1) providing support to a person over the age of 65, 2) providing assistance for more than one month, 3) not receiving any financial compensation, 4) being over the age of 18, and 5) providing assistance on a daily basis. The formal caregivers and the relatives providing assistance for the last few days before hospitalization or providing assistance occasionally were excluded.

For the purpose of the study, two separate forms were used, one for the patients and a second for their caregivers. Caregivers' forms were self-administered. Caregivers who agreed to participate were asked to fill out a questionnaire including information regarding their age, sex, marital status, educational level, relationship with the care recipient, cohabitation with the care recipient, employment, duration of caregiving, hours of caregiving per day, and chronic medical problems when the patients were admitted. The caregivers' form included the translated and validated in Greek [12] Zarit Burden Interview (ZBI) [13] to assess subjective caregivers' burden and the translated and the validated in the Greek language [14] Hospital Anxiety and Depression Scale (HADS) [15] to assess caregivers' anxiety. The questionnaires were given to the caregivers, and they were picked up later. The average time for questionnaire completion was twenty minutes. After the completion of the questionnaire, the authors checked for unanswered questions, and if there were any, they kindly requested the caregivers to fill them up. Later, the authors calculated the total scores for the ZBI and HADS. A Charlson Comorbidity Index (CCI) [16] for the caregivers was calculated based on caregivers' personal health problems.

A second interview-based form was used to collect information about patients' demographics (age, gender, educational level, marital status), medical and medication history (comorbidities, number and type of medications), body mass index (BMI), the reason for admission, frailty assessment, cognitive status assessment, the presence of behavioral problems, and dependency on activities of daily living. Patients were asked about their demographics, medical and medication history, and functionality. When patients were unable to communicate, their caregivers provided information. Frailty status, cognitive status, CCI, and dependency on activities of daily living were assessed for each patient by the researchers. The average time for patients' questionnaire completion was thirty minutes.

Instruments/tools

The ZBI is a 22-item instrument created by Zarit et al. [13] to assess caregivers' burden. The caregiver's emotional wellbeing, interpersonal relationships, financial status, and interaction with the care recipient are all reflected in the 22 questions. The potential answers are rated from 0 to 4 and represent the frequency with which the caregiver felt a certain way (0=never, 1=rarely, 2=sometimes, 3=frequently, and 4=nearly always). The overall score, which ranges from 0 to 88, is calculated by summing the values of each response. The greater the caregiver burden, the higher the score. Additionally, caregiver burden can be classified into four categories based on the ZBI score: 1) little or no burden (score 0-20), 2) mild to moderate burden (score 21-40), 3) moderate to severe burden (score 41-60), and 4) severe burden (score 61-88) [13]. An objective burden was considered to be determined by the care needs of the dependent elderly patients (frailty status, cognitive impairment, comorbidity, independence in activities of daily living, behavioral problems, hours spent for caregiving, and duration on caregiving). The Hospital Anxiety and Depression Scale (HADS) is a reliable, valid, and easy-to-use tool that was originally developed for identifying and quantifying patients in need of further psychiatric evaluation and assistance [15]. The Charlson Comorbidity Index (CCI) is a measure that includes most major medical comorbidities. It consists of 17 comorbidity categories, including age categories, and each one has an associated score (from 1 to 6). The sum of individual weights for each patient results in a total comorbidity score [16].

Frailty was assessed using the revised nine-scale Clinical Frailty Scale (CFS) [17], which was translated and validated in Greek [18]. The CFS is a judgment-based frailty tool that summarizes the overall level of fitness or frailty of an older adult to generate a frailty score ranging from 1 (very fit) to 9 (terminally ill) [17]. The Barthel Index (BI) was used to assess the performance in activities of daily living. The BI is an ordinal scale ranging from 0 to 100, and it measures functional independence in ten domains of personal care and mobility [19]. The Global Deterioration Scale (GDS) [20] was used to assess cognitive status. The GDS is a seven-point scale that ranges from 1 (no cognitive decline) to 7 (very severe cognitive decline - severe dementia). The presence of behavioral problems was assessed by using the question: has the patient presented delirium and/or behavioral disorders requiring antipsychotic drugs in the last six months? Based on information obtained from the patients and/or their caregivers and the patients' medical history, CFS, BI, GDS, and CCI were estimated for the patients' baseline status prior to the onset of acute illness that led to seeking medical assistance.

Ethical considerations

Τhe study was designed by the Department of Health Economics of the School of Health Sciences of the National and Kapodistrian University of Athens and implemented in the Department of Internal Medicine of General and Oncology Hospital of Kifissia "Agioi Anargyroi". The Institutional Ethical and Scientific Committee of General and Oncology Hospital of Kifissia "Agioi Anargyroi" (approval number: 1494; date of approval: 04/12/2019) and the Committee on Bioethics and Deontology of School of Medicine, National and Kapodistrian University of Athens (approval number: 284; date of approval: 25/05/2020) both approved the study. Informed written consent was obtained from both the patient and their caregivers, or only from the caregiver when the patient could not communicate. The forms for both patients and caregivers were accompanied by a cover letter explaining the purpose of the study on the first page. Furthermore, it was mentioned on the first page that the final data reports would maintain confidentiality and anonymity.

Statistical analyses

**Figure 1:**
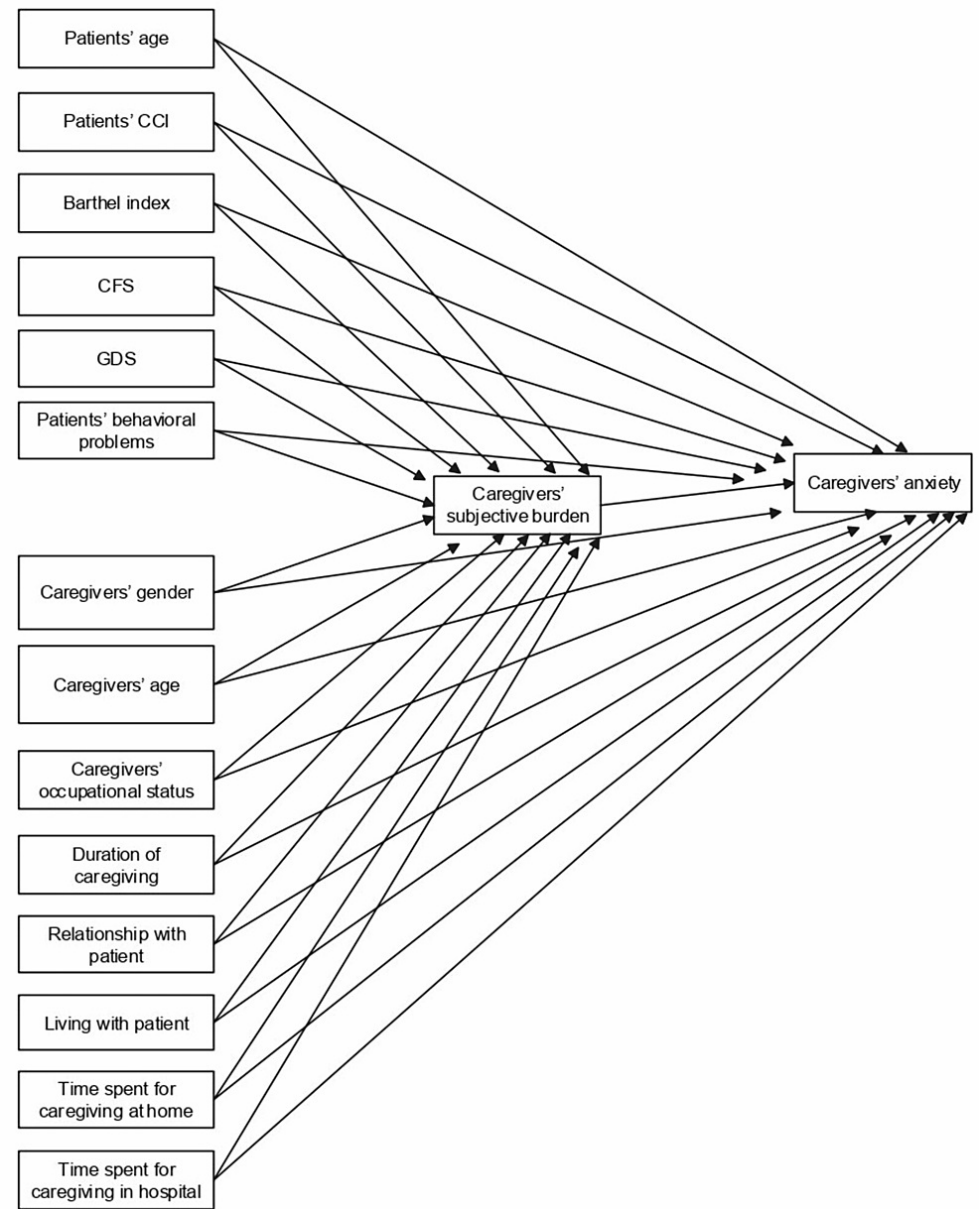
Initial saturated model with caregivers' anxiety as the outcome variable All paths were declared to be optional. Covariances among exogenous variables have been omitted for simplicity. CFS - Clinical Frailty Scale; CCI - Charlson Comorbidity Index; GDS - Global Deterioration Scale

Descriptive statistics were computed for socio-demographic, psychological, and medical variables. To assess monotonic relationships between variables, Spearman's rank correlation coefficients were used. We computed the total sample size required to determine whether a small to moderate correlation coefficient (effect size of 0.20) differed from zero, given a 0.05 significance level and 0.90 statistical power. The minimum sample size was estimated to be 258. Medians were reported in the analysis. Initially, an exploratory specification search using Amos 23 [21] was performed, with 29 optional regression paths between 16 observed variables, to compare competing models involving all possible combinations of paths and identify the best fitting model (Figure 1).

Path analysis enables researchers to decompose relations among variables into direct, indirect, and spurious components (representing all unmeasured causes on which endogenous variables may depend) so that the significant pathways involved in predicting an outcome can be identified. Caregivers' subjective burden was entered as a mediator, while caregivers' anxiety was considered as the outcome variable. Variables hypothesized to be explained by others were endogenous variables, while those not predicted by others (e.g., duration of caregiving, CFS) were exogenous variables.

Multivariate outliers were checked by examining Mahalanobis distances. Multivariate normality was checked by applying Mardia's test for multivariate kurtosis. Applying the maximum likelihood method, parameter estimates were derived. Bootstrapping, a non-parametric method based on resampling with replacement, was conducted, generating 5,000 bootstrap samples, and the 95% bias-corrected bootstrap confidence intervals (CI) for indirect, total, and direct effects were estimated. The following four indices were used to assess the goodness of fit of the model: the root mean square error of approximation (RMSEA) accompanied by its associated 90% confidence interval, the standardized root mean square residual (SRMR), the comparative fit index (CFI), and the Tucker-Lewis Index (TLI). When CFI and TLI were greater than 0.95, SRMR was less than 0.08, and RMSEA was less than 0.06 [22], model fit was deemed adequate [22]. Improvements to model fit were indicated by a decrease in the model Akaike information criterion (AIC) and the model Bayesian information criterion (BIC).

## Results

During the study period, 504 elderly patients were admitted to the medical unit via the emergency department. Totally, 182 of the participants were excluded due to the non-availability of the caregiver and 11 due to non-consent (response rate 96.6%). Finally, 311 patient-caregiver dyads were enrolled in the study. The median age of caregivers was 56 years old (IQR: 49-65). Among the caregiving participants, 232 were females (74.6%), and 79 were males (25.4%). The median CCI of caregivers was 1.00 (IQR: 1.00-2.00). Regarding ZBI scores, 72 caregivers (23.2%) were classified as having little or no burden (score 0-20), 113 (36.3%) as having mild to moderate burden (score 21-40), 100 (32.2%) as having moderate to severe burden (score 41-60), and 26 (8.4%) as having severe burden (score 61-88). Regarding HADS scores, 153 caregivers (49.2%) were classified as normal (score 0-7), 66 (21.2%) as borderline abnormal (score 8-10), and 92 (29.6%) as abnormal (score 11-21). Caregivers' characteristics are presented in Table 1.

**Table 1:** Caregivers' characteristics (n=311) IQR - interquartile range

Caregivers' characteristics	n (%)
Gender	
Males	79 (25.4%)
Females	232 (74.6%)
Age (years; median-IQR)	56.00 (49.00-65.00)
Marital status	
Married	205 (65.9%)
Unmarried	49 (15.8%)
Divorced	44 (14.1%)
Widowed	13 (4.2%)
Educational status	
Primary	59 (19.0%)
Secondary	111 (35.7%)
Technological education institution	66 (21.2%)
University	75 (24.1%)
Living with the patient	
Yes	190 (61.1%)
No	121 (38.9%)
Relationship with the patient	
Spouse/partner	75 (24.1%)
Son/daughter	196 (63.0%)
Other	40 (12.9%)
Employment	
Employed	164 (52.7%)
Unemployed	56 (18.0%)
Retired	91 (29.3%)
Hours per day spent for caregiving at home (median-IQR)	5.00 (2.00-12.00)
Hours per day spent for caregiving at hospital (median-IQR)	8.00 (4.00-12.00)
Duration of caregiving (in months; median-IQR)	24.00 (6.00-60.00)

The median age of patients was 84 years (IQR: 78-89), while 152 were females (48.9%) and 159 were males (51.1%). Patients' characteristics are presented in Table 2.

**Table 2:** Patients' characteristics (n=311) IQR - interquartile range; CCI - Charlson Comorbidity Index; BI - Barthel Index; GDS - Global Deterioration Scale; CFS - Clinical Frailty Scale

Patients' characteristics	n (%)
Gender	
Males	159 (51.1%)
Females	152 (48.9%)
Age (years; median-IQR)	84.00 (78.00-89.00)
CCI (median-IQR)	6.00 (5.00-7.00)
Behavioral problems	
Yes	62 (19.9%)
No	249 (80.1%)
Marital status	
Married	152 (48.9%)
Unmarried	5 (1.6%)
Divorced	5 (1.6%)
Widowed	149 (47.9%)
Educational status	
Primary	189 (60.8%)
Secondary	80 (25.7%)
Technological education institution	31 (10.0%)
University	11 (3.5%)
BI (median-IQR)	70.00 (35.00-95.00)
GDS score (median-IQR)	1.00 (0.00-3.00)
CFS score (median-IQR)	6.00 (4.00-7.00)

Table 3 shows the prevalence of the most common specific patients' comorbidities. Seven out of fifteen comorbidities are not included in Charlson Comorbidity Index.

**Table 3:** Prevalence of the most frequent specific comorbidities *Comorbidities that were not included in Charlson Comorbidity Index. COPD - chronic obstructive pulmonary disease

Comorbidities	n	Percentage
Arterial hypertension*	184	59.2%
Diabetes mellitus	98	31.5%
Hyperlipidemia*	84	27.0%
Atrial fibrillation*	77	24.8%
Dementia	76	24.5%
Cancer	72	23.2%
Heart failure	61	19.6%
Hypothyroidism*	54	17.4%
COPD	53	17.0%
Coronary artery disease	48	15.4%
Depression-anxiety*	41	13.1%
Benign prostate hyperplasia*	38	12.2%
Stroke	34	10.9%
Chronic renal failure	28	9.0%
Parkinson's disease*	27	8.7%

Correlations among variables

Table 4 presents Spearman's correlation coefficients between the main psychological, socio-demographic, and medical variables included in the analyses. Higher caregivers' anxiety was significantly correlated with higher caregivers' burden (rho=0.555, p<0.01), higher CFS (rho=0.292, p<0.01), longer duration of caregiving (rho=0.209, p<0.01), and lower Barthel Index (rho=-0.237, p<0.01). Among the remaining correlations, there was a noteworthy correlation between caregivers' burden, CFS (rho=0.434, p<0.01), and duration of caregiving (rho=0.436, p<0.01).

**Table 4:** Spearman's correlation coefficients between selected predictor, mediator, and outcome variables (n=311) *p < 0.05, **p < 0.01 CFS - Clinical Frailty Scale; CCI - Charlson Comorbidity Index; GDS - Global Deterioration Scale

Variable	1	2	3	4	5	6
1. Caregivers' anxiety						
2. Caregivers' burden	0.555**					
3. CFS	0.292**	0.434**				
4. Patients' CCI	0.078	0.138*	0.427**			
5. Duration of caregiving	0.209**	0.436**	0.326**	0.076		
6. Barthel index	-0.237**	-0.379**	-0.669**	-0.151**	-0.377**	
7. GDS	0.100	0.244**	0.398**	0.053	0.235**	-0.633**

Path analysis

During the exploratory specification search using Amos 23, 14 variables were considered exogenous predictors (patients' age, patients' CCI, Barthel Index, CFS, GDS, patients' behavioral problems, caregivers' gender, caregivers' age, caregivers' occupational status, duration of caregiving, relationship with patient, living with patient, time spent for caregiving at home, time spent for caregiving in hospital), while two observed variables (caregivers' subjective burden, caregivers' anxiety) were regarded as endogenous. This model was a saturated one, where no constraints were placed on the population moments (variances and covariances). Covariances among the exogenous variables were allowed. The saturated model had χ^2^=0, zero degrees of freedom, 136 distinct parameters to be estimated, normed fit index (NFI) of 1, CFI of 1, goodness to fit (GFI) statistic of 1, SRMR of 0, and RMSEA of 0.205. Given that an exhaustive search would require fitting a vast number of candidate models (actually, 2^29^=536,870,912 models), which was not computationally feasible, a stepwise heuristic approach was adopted to reduce the number of models that had to be fitted. The stepwise procedure added or removed a path only if the resulting model had a smaller discrepancy than any previously encountered model with the same number of paths. The algorithm fitted 434 models per pass by constraining the saturated model and using every subset of the optional paths between variables. Arranging fitted models by descending order of values regarding the discrepancy function C (which is the likelihood ratio chi-square statistic), Table 5 presents the top 10 models for each number of parameters (i.e., for each combination of paths) from a shortlist of 30 best-fitting models. Beyond model 10, increasing the number of parameters corresponded to small improvements in C.

**Table 5:** Top 10 best models according to stepwise specification search for increasing number of parameters and different fit measures to compare models df - degrees of freedom; C - discrepancy function (likelihood ratio Chi-square statistic); AIC_0_ - Akaike information criterion rescaled so that the smallest value is zero; BIC_0_ - Bayesian information criterion rescaled so that the smallest value is zero; BIC_L_- Bayes factors rescaled so that the largest value is 1 and can be interpreted as a likelihood

Models	Number of parameters	df	C	C/df	AIC_0_	BIC_0_	BIC_L_
1	107	29	271.68	9.37	242.67	216.62	0
2	108	28	152.94	5.46	125.93	103.63	0
3	109	27	86.02	3.19	61.01	42.45	0
4	110	26	43.82	1.69	20.81	5.99	0.05
5	111	25	33.20	1.33	12.19	1.11	0.58
6	112	24	26.36	1.10	7.35	0	1
7	113	23	21.56	0.94	4.55	0.94	0.62
8	114	22	17.33	0.79	2.32	2.45	0.29
9	115	21	14.15	0.67	1.14	5.02	0.08
10	116	20	11.42	0.57	0.41	8.02	0.02

**Figure 2:**
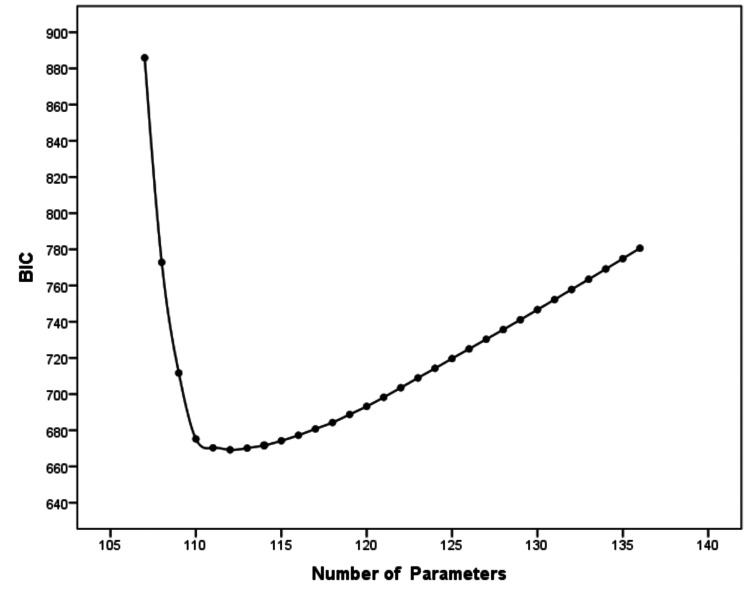
Best-fit plot for models with different number of parameters, based on the BIC fit measure An "elbow" in the graph provides support for the best model. BIC - Bayesian information criterion

To compare models, AIC_0_ and BIC_0 _values were used (i.e., AIC and BIC values that had been rescaled so that the optimal value was 0). AIC_0_ values between 0 and 2 could be interpreted as suggesting that there was no credible evidence that a model should be ruled out as being the actual best model. Values 2-4 denoted that there was weak evidence that a model was not the best model, whereas values >4 suggested that there was definite or strong evidence that the model was not the best one. According to AIC_0_, models 8, 9, and 10 should not be ruled out as being the best models for the population of possible samples. Regarding BIC_0_, values ranging from 0 to 2 indicated weak evidence against a model assumed to be the best model, values 2-6 suggested positive evidence against a model, while values >6 implied strong or very strong evidence against a model as compared to other better-competing models. According to BIC_0_, model 6, with 112 parameters and 24 degrees of freedom, was the best model (BIC_0_=0). The second-best model was model 7, with 113 parameters and 23 degrees of freedom (BIC_0_=0.944), followed by model 5 (BIC_0_=1.108) and model 8 (BIC_0_=2.448). Inspection of the BIC_L_ values (Akaike weights using Bayes factors rescaled so that the largest value was 1) revealed that model 6 had the best value (BIC_L_=1), followed by model 7 (BIC_L_=0.624), model 5 (BIC_L_=0.575), and model 8 (BIC_L_=0.294). Viewing the best-fit graph for BIC (a fit measure that imposed a great penalty for complexity) made it clear that model 8, with 114 parameters and 22 degrees of freedom, was superior to the other candidate models. As the number of parameters increased beyond 114, a steep positive slope in the graph appeared (Figure 2).

**Figure 3:**
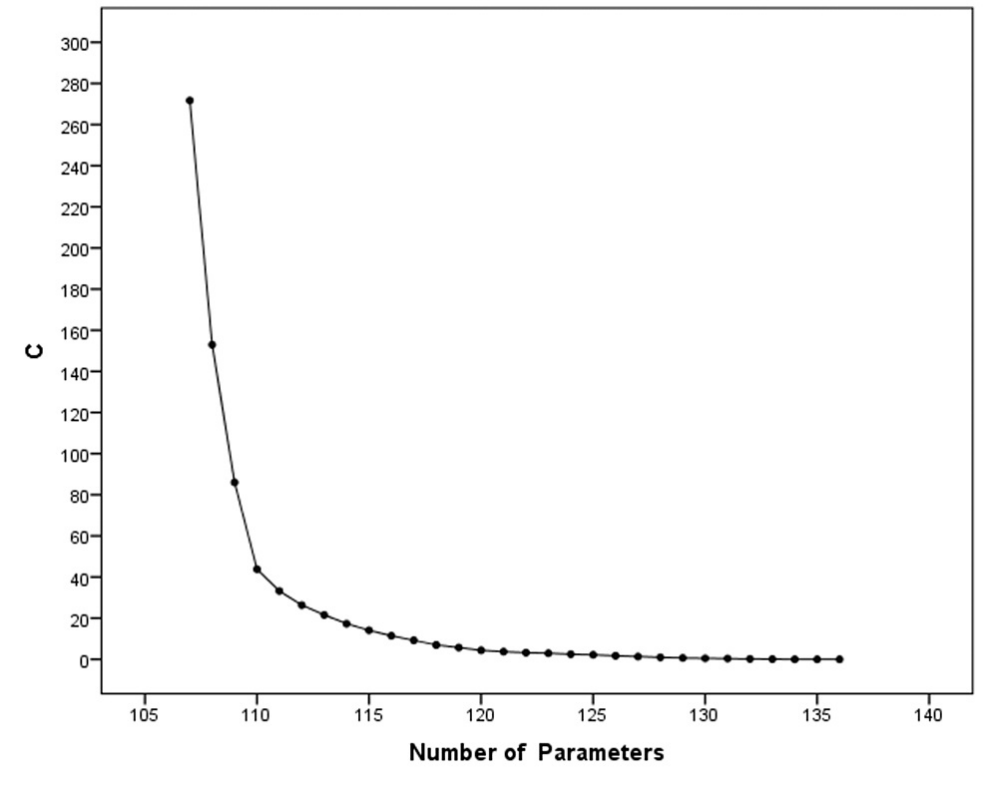
Scree plot for models with different number of parameters, based on the C fit measure A point in the graph beyond which the slope becomes relatively flat provides support for the best model. C - discrepancy function

Additionally, viewing the scree plot for C, we could notice that the height of the graph at 115 parameters represented the improvement in C obtained by moving from the 114-parameter model (model 8) to the 115-parameter model (model 9). The slope changed at 115 parameters and became relatively flat, a change that provided support for the previous 114-parameter model 8 (Figure 3).

**Figure 4:**
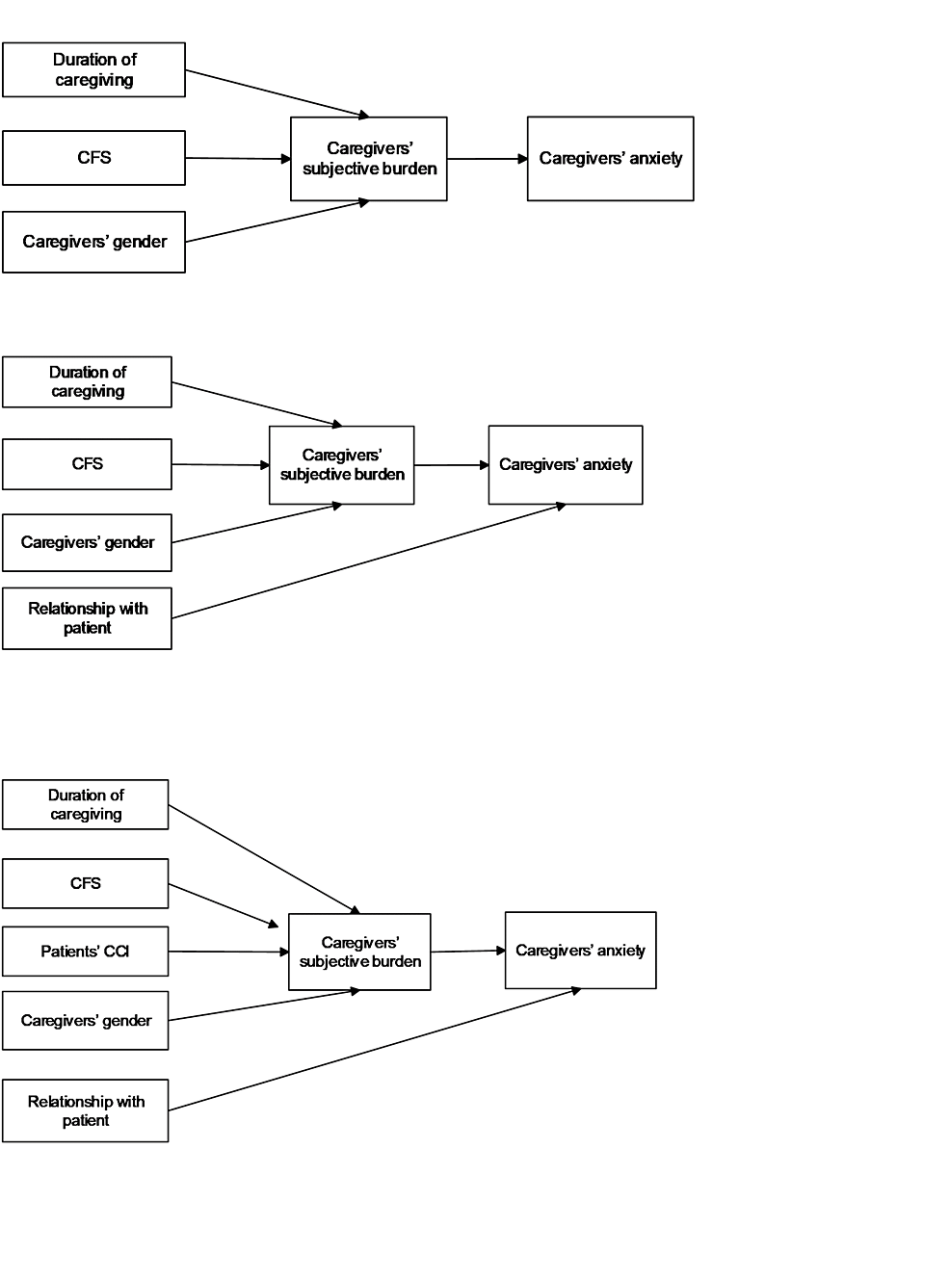
Competing models 5, 6, and 7, with 111, 112, and 113 parameters to be estimated, respectively CFS - Clinical Frailty Scale; CCI - Charlson Comorbidity Index

This heuristic, called the "point of diminishing returns", testified to the superiority of model 8. Moreover, beyond model 8, increasing the number of parameters yielded nonsignificant improvements in C (<3.84). Based on the above analysis, model 8 emerged as the best-chosen model from among the competing models (Figure 4).

Final path model

**Figure 5:**
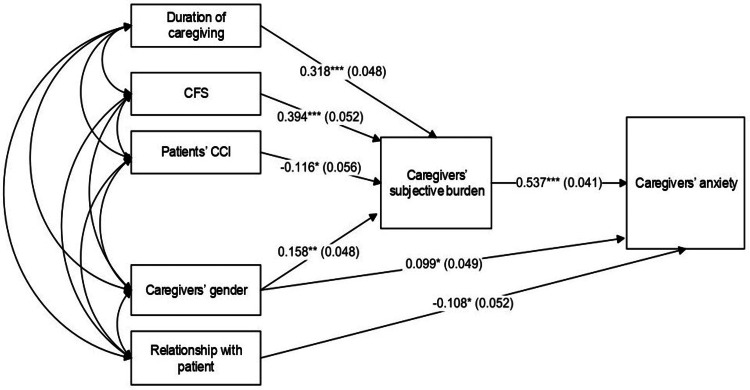
Standardized direct path coefficients (and their standard errors) for the final model (model 8) obtained from path analysis with observed variables (n=311) *p<0.05, **p<0.01, ***p<0.001 CFS - Clinical Frailty Scale; CCI - Charlson Comorbidity Index

Figure 5 shows model 8 with 28 sample moments (the variances and covariances of the observed variables, from which the model parameters are derived) and 24 parameters to be estimated (unknown population path coefficients, exogenous variable variances and covariances, and residual error variances).

Given that path models assumed a multivariate normal distribution, we assessed joint multivariate kurtosis and checked outliers. Although there were separate variables with skewness or kurtosis values suggestive of non-normality in the sample, the multivariate kurtosis value was small (equal to -0.847) and was considered negligible and nonsignificant at the 0.05 level, based on the critical ratio criterion (equal to -0.665<1.96). No cases with large Mahalanobis distance values were detected, signifying that no outliers existed and no cases were located farthest from the centroid of the data set. This model had a satisfactory fit to the data, with χ^2^(4)=6.185, p=0.186, TLI=0.968, CFI=0.994, RMSE=0.042 (90% CI: 0.000-0.103), SRMR=0.022. NFI of 0.984 indicated that the model had a discrepancy that was 98.4%, close to the perfectly fitting saturated model and far away from the worst fitting independence model. Furthermore, based on the relatively small (<2.58) standardized residual covariances, this model was a good fit and could reproduce the original variance-covariance matrix from the path coefficients adequately. The variables included in the path analysis accounted for a satisfactory proportion of the variance in caregivers' subjective burden (33.1%) and for an equivalent proportion of the variance in caregivers' anxiety (33.6%). The remaining 66.9% and 66.4% of these variances, respectively, represented measurement error and error due to other factors on which caregivers' burden or caregivers' anxiety might depend but which were not measured in this study.

Direct effects in path analysis 

As can be seen in Figure 5, caregivers' subjective burden was supposed to be a mediator in the relationship between the exogenous variables and caregivers' anxiety. Three of the standardized regression coefficients pertaining to direct effects among study variables achieved statistical and practical significance (the latter meaning that beta weights were above 0.30 in absolute value, signifying at least a medium effect size), while four other path coefficients were significant but of relatively lower magnitude. In particular, caregivers' subjective burden was significantly related to higher caregivers' anxiety (β=0.537, p<0.001), duration of caregiving was related to higher caregivers' subjective burden (β=0.318, p<0.001), and CFS was associated with higher caregivers' burden (β=0.394, p<0.001). Patients' CCI was negatively related to caregivers' subjective burden (β=-0.116, p<0.05). Moreover, the caregivers' female gender had a positive and statistically significant direct path to caregivers' subjective burden (β=0.158, p<0.01) and a positive direct path to caregivers' anxiety (β=0.099, p<0.05). Finally, being a patient's spouse was associated with greater caregiver anxiety (β=-0.108, p<0.05) than being the patient's child or sibling.

Total effects in mediation analysis

Regarding noteworthy significant standardized total effects, duration of caregiving (effect=0.171, 95% CI: 0.117-0.231), CFS (effect=0.212, 95% CI: 0.153-0.286), and caregivers' female gender (effect=0.183, 95% CI: 0.072-0.290) were positively related to caregivers' anxiety. Patients' CCI (effect=-0.062, 95% CI: -0.125 - -0.006) and being a child/sibling of a patient (effect=-0.108, 95% CI: -0.210 - -0.006) had significant standardized total effects on lower caregivers' anxiety. All these 95% confidence intervals did not contain zero, suggesting that the corresponding total effects were statistically significant at the 0.05 level.

Indirect effects in mediation analysis

As shown in Table 6, regarding significant two-path standardized indirect effects, CFS, duration of caregiving, and female caregivers' gender were all significantly related to higher caregivers' anxiety through caregivers' subjective burden. Furthermore, patients' CCI was significantly and negatively associated with caregivers' anxiety via caregivers' burden.

**Table 6:** Standardized parameter estimates and 95% bootstrapped confidence intervals for indirect effects *p<0.05, **p<0.01, ***p<0.001 SE - standard error; CI - confidence interval; LL - lower limit; UL - upper limit; CFS - Clinical Frailty Scale; CCI - Charlson Comorbidity Index

Predictor variable		Mediator		Outcome variable	Parameter estimate (SE)	95% CI	
						LL	UL
CFS	→	Caregivers' burden	→	Caregivers' anxiety	0.212*** (0.034)	0.153	0.286
Duration of caregiving	→	Caregivers' burden	→	Caregivers' anxiety	0.171*** (0.029)	0.117	0.231
Caregivers' gender	→	Caregivers' burden	→	Caregivers' anxiety	0.085** (0.026)	0.036	0.138
Patients’ CCI	→	Caregivers' burden	→	Caregivers' anxiety	-0.062* (0.031)	-0.125	-0.006

These findings emphasized the central mediating role of caregivers' burden in the relationship between patients' and caregivers' characteristics and caregivers' anxiety. More importantly, as shown by the noteworthy indirect (mediated) effects, although CFS did not have significant direct effects on caregivers' anxiety (β=0.076, SE=0.050, p>0.05), it did have a significant indirect effect on this latter variable.

## Discussion

In the present study, we found that the female gender of the caregiver, the spousal relationship with the patient, and the subjective burden (ZBI score) were directly related to higher levels of caregivers' anxiety. Moreover, subjective burden mediated the effects of duration of caregiving, patients' frailty status, caregiver gender, and patients' comorbidity on caregivers' anxiety.

The effect of subjective caregiver burden on anxiety was highlighted in a systematic review [6], in a systematic review and meta-analysis [7], and in a study that used path analysis in order to identify the effects of several variables on caregivers' anxiety [8]. Furthermore, previous research has linked the female gender to higher anxiety levels [2,4] and greater subjective burden [9]. Likewise, spousal relationships with care recipients were related to higher caregivers' anxiety levels [2]. The duration of caregiving has been reported previously as a risk factor for caregivers' burden [23] but not as a risk factor for caregivers' anxiety. Finally, caregivers' burden has been previously reported to be higher when patients' comorbidity increases [24], but as far as we know, no study has associated patients' comorbidity with caregivers' anxiety. In our study, we found that patients' CCI was significantly and negatively associated with caregivers' anxiety via caregivers' burden. In our opinion, this result has two possible explanations. The CCI was originally developed as a weighted index for patients with specific comorbid conditions to predict the risk of mortality within one year of hospitalization. As mentioned before, it consists of 17 comorbidity categories, including age categories, and each one has an associated score (from 1 to 6). The sum of individual scores for each patient results in a total comorbidity score [16]. However, CCI is not a measure of the overall health status or a measure of multimorbidity [25]. Indeed, in our sample, seven out of fifteen concomitant morbidities were not included in CCI, perhaps biasing the specific effect of patients' comorbidity on caregivers' burden or anxiety. Furthermore, a study sample characteristic that could bias the specific effect of patients' comorbidity on caregivers' burden or anxiety might be that almost one out of four patients had a history of cancer (metastatic or not), a condition with a high associated weight in CCI. Burden or anxiety levels of the caregiver of a patient with metastatic cancer (resulting in a high CCI) and good performance status may indeed be lower than that of a caregiver of a bedridden patient suffering from Parkinson's disease, hypertension, dyslipidemia, and hypothyroidism (resulting in a low CCI). An additional explanation would be that caregiving might have positive aspects as well. For example, caregiving may be associated with a sense of role fulfillment and personal accomplishments, feelings of personal purpose, meaning, and growth, an increase in family cohesion, and a sense of mutuality in the dyadic relationship. These positive aspects of caregiving for elderly patients with comorbidities may reduce levels of caregivers' burden [26]. Regarding the effect of frailty status on caregivers' anxiety, most previous studies did not include frailty measures among the evaluated variables, so no data for comparison was available. On the other hand, frailty is a known risk factor for caregivers' burden [27].

Identifying the factors that are directly or indirectly associated with caregiver anxiety is the first step toward addressing non-pharmacological interventions to reduce caregiver anxiety levels [28]. Duration of caregiving, caregivers' gender, the relationship with the patient, and patients' comorbidity are all non-modifiable risk factors. On the other hand, interventions to reduce the subjective caregivers' burden have been applied, resulting in positive effects for a short period [29]. Maybe interventions targeted to reduce or reverse frailty, such as muscle strength training and protein supplementation [30], could be more efficient in controlling caregivers' anxiety.

Strengths and limitations

We are aware of the limitations of our study. First, the cross-sectional design does not allow us to infer causal relationships or directionality. Longitudinal studies are needed to understand the sequence and causal nature of the relationships between variables. Second, the assessment of caregivers' anxiety was conducted during a stressful event such as the hospitalization of their loved ones. However, we focused only on caregivers of elderly hospitalized patients who had already been involved in the caregiving process before hospitalization, considering that caregiving is a continuous process that has a dynamic nature over time and a stressful event such as hospitalization is another link in the chain of this process. Third, given that a satisfactory yet moderate proportion of variance in endogenous variables was successfully accounted for by the variables included in our model, future studies should consider including more variables that may influence caregivers' anxiety, such as personality factors, social support, and coping strategies. Fourth, models and results derived from exploratory specification searches are data-driven and are likely to be influenced by chance characteristics of the sample. Although we confirmed our results by conducting mediation analysis using Hayes' PROCESS program, cross-validation studies are needed to fit such models to independent samples. Finally, obtaining data from both members of the patient-caregiver dyad may violate the assumption of independence, given that the responses of the carer and the care recipient are nested in the dyad and are hierarchical in nature, so that observations from individuals within the same dyad are more likely similar and non-independent. In addition, both members of the dyad are deliberately sampled to study the influence they may have on one another, thus violating the assumption of random sampling from the population. Future research should use multilevel modeling and actor-partner interdependence models (APIM) to analyze data collected from patients and their caregivers.

The study's strength is that it evaluates caregivers' anxiety in a sample of caregivers of patients who do not belong to a disease-specific group but rather in a sample that includes a broad spectrum of diseases and comorbidities. Second, it has included several measures that describe globally the status of elderly patients (frailty, mental health, comorbidity, independence in activities of daily living), as possible factors that may be associated with caregivers' anxiety and burden.

## Conclusions

We can conclude that in family caregivers of dependent elderly people, the female gender of the caregiver, the spousal relationship with the patient, and the subjective caregivers' burden are risk factors for caregivers' anxiety. Moreover, caregivers' subjective burden mediates the effects of duration of caregiving, patients' frailty status, caregiver gender, and patients' comorbidity on caregivers' anxiety. Finally, among the risk factors for caregivers' anxiety, probably the only modifiable risk factor is the frailty status of the patient, with interventions aimed at reducing or reversing frailty.

## References

[1] Schulz R, Beach SR (1999). Caregiving as a risk factor for mortality: the caregiver health effects study. JAMA.

[2] Sallim AB, Sayampanathan AA, Cuttilan A, Ho R (2015). Prevalence of mental health disorders among caregivers of patients with Alzheimer disease. J Am Med Dir Assoc.

[3] Felipe SG, Oliveira CE, Silva CR, Mendes PN, Carvalho KM, Lopes Silva-Júnior F, Figueiredo MD (2020). Anxiety and depression in informal caregivers of dependent elderly people: an analytical study. Rev Bras Enferm.

[4] Mahoney R, Regan C, Katona C, Livingston G (2005). Anxiety and depression in family caregivers of people with Alzheimer disease: the LASER-AD study. Am J Geriatr Psychiatry.

[5] Liang X, Guo Q, Luo J, Li F, Ding D, Zhao Q, Hong Z (2016). Anxiety and depression symptoms among caregivers of care-recipients with subjective cognitive decline and cognitive impairment. BMC Neurol.

[6] Cooper C, Balamurali TB, Livingston G (2007). A systematic review of the prevalence and covariates of anxiety in caregivers of people with dementia. Int Psychogeriatr.

[7] Del-Pino-Casado R, Priego-Cubero E, López-Martínez C, Orgeta V (2021). Subjective caregiver burden and anxiety in informal caregivers: a systematic review and meta-analysis. PLoS One.

[8] Pérez-Cruz M, Parra-Anguita L, López-Martínez C, Moreno-Cámara S, Del-Pino-Casado R (2019). Burden and anxiety in family caregivers in the hospital that debut in caregiving. Int J Environ Res Public Health.

[9] Adelman RD, Tmanova LL, Delgado D, Dion S, Lachs MS (2014). Caregiver burden: a clinical review. JAMA.

[10] Pearlin LI, Mullan JT, Semple SJ, Skaff MM (1990). Caregiving and the stress process: an overview of concepts and their measures. Gerontologist.

[11] Spillman BC, Wolff J, Freedman VA, Kasper JD (2022). Informal caregiving for older Americans: an analysis of the 2011 National Study of Caregiving. https://aspe.hhs.gov/reports/informal-caregiving-older-americans-analysis-2011-national-study-caregiving.

[12] Parpa E, Katsantonis NG, Tsilika E, Galanos A, Papazoglou I, Mystakidou K (2017). Validity and reliability of the Greek version of the ZBI in informal carers of adults with intellectual disabilities. Int J Ment Health Psychiatry.

[13] Zarit SH, Reever KE, Bach-Peterson J (1980). Relatives of the impaired elderly: correlates of feelings of burden. Gerontologist.

[14] Michopoulos I, Douzenis A, Kalkavoura C (2008). Hospital Anxiety and Depression Scale (HADS): validation in a Greek general hospital sample. Ann Gen Psychiatry.

[15] Zigmond AS, Snaith RP (1983). The hospital anxiety and depression scale. Acta Psychiatr Scand.

[16] Deyo RA, Cherkin DC, Ciol MA (1992). Adapting a clinical comorbidity index for use with ICD-9-CM administrative databases. J Clin Epidemiol.

[17] Pulok MH, Theou O, van der Valk AM, Rockwood K (2020). The role of illness acuity on the association between frailty and mortality in emergency department patients referred to internal medicine. Age Ageing.

[18] Vrettos I, Voukelatou P, Panayiotou S (2021). Validation of the revised 9-scale clinical frailty scale (CFS) in Greek language. BMC Geriatr.

[19] Wade DT, Collin C (1988). The Barthel ADL Index: a standard measure of physical disability?. Int Disabil Stud.

[20] Reisberg B, Ferris SH, de Leon MJ, Crook T (1982). The Global Deterioration Scale for assessment of primary degenerative dementia. Am J Psychiatry.

[21] Arbuckle JL (2014). Amos (version 23.0) [computer program].

[22] Hu L, Bentler PM (1999). Cutoff criteria for fit indexes in covariance structure analysis: conventional criteria versus new alternatives. Struct Equ Model.

[23] Ransmayr G, Hermann P, Sallinger K (2018). Caregiving and caregiver burden in dementia home care: results from the prospective dementia registry (PRODEM) of the Austrian Alzheimer Society. J Alzheimers Dis.

[24] Dauphinot V, Ravier A, Novais T (2016). Relationship between comorbidities in patients with cognitive complaint and caregiver burden: a cross-sectional study. J Am Med Dir Assoc.

[25] Charlson ME, Carrozzino D, Guidi J, Patierno C (2022). Charlson Comorbidity Index: a critical review of clinimetric properties. Psychother Psychosom.

[26] Yu DSF, Cheng ST, Wang J (2018). Unravelling positive aspects of caregiving in dementia: An integrative review of research literature. Int J Nurs Stud.

[27] Ringer T, Hazzan AA, Agarwal A, Mutsaers A, Papaioannou A (2017). Relationship between family caregiver burden and physical frailty in older adults without dementia: a systematic review. Syst Rev.

[28] Sun Y, Ji M, Leng M, Li X, Zhang X, Wang Z (2022). Comparative efficacy of 11 non-pharmacological interventions on depression, anxiety, quality of life, and caregiver burden for informal caregivers of people with dementia: a systematic review and network meta-analysis. Int J Nurs Stud.

[29] Mou H, Wong MS, Chien WT (2021). Effectiveness of dyadic psychoeducational intervention for stroke survivors and family caregivers on functional and psychosocial health: a systematic review and meta-analysis. Int J Nurs Stud.

[30] Travers J, Romero-Ortuno R, Bailey J, Cooney MT (2019). Delaying and reversing frailty: a systematic review of primary care interventions. Br J Gen Pract.

